# 2892. Safety and Immunogenicity of mRNA-1647, an mRNA-Based Cytomegalovirus Vaccine in Healthy Adults: Results of a Phase 2, Randomized, Observer-Blind, Placebo-Controlled, Dose-Finding Trial

**DOI:** 10.1093/ofid/ofad500.2475

**Published:** 2023-11-27

**Authors:** Lori Panther, Sandeep Basnet, Carlos Fierro, Daniel Brune, Richard Leggett, James Peterson, Paul Pickrell, Jiang Lin, Kai Wu, Heather Lee, Roxane Hasselbeck, Andrew Natenshon, Jacqueline Miller

**Affiliations:** Moderna, Inc., Cambridge, Massachusetts; Moderna, Inc., Cambridge, Massachusetts; Johnson County Clin-Trials, Lenexa, Kansas; Optimal Research, Peoria, Illinois; Crossroads Clinical Research, Victoria, Texas; Foothill Family Clinic, Salt Lake City, Utah; Tekton Research, Austin, Texas; Moderna, Inc., Cambridge, Massachusetts; Moderna, Inc., Cambridge, Massachusetts; Moderna, Inc., Cambridge, Massachusetts; Moderna, Inc., Cambridge, Massachusetts; Moderna, Inc., Cambridge, Massachusetts; Moderna, Inc., Cambridge, Massachusetts

## Abstract

**Background:**

A safe and effective method to protect against cytomegalovirus (CMV) infection is a public health priority. mRNA-1647 is an investigational mRNA-based vaccine against CMV consisting of 6 mRNA sequences encoding 2 CMV antigens (glycoprotein B and the pentameric glycoprotein complex) in lipid nanoparticles in a lyophilized presentation.

**Methods:**

This phase 2, randomized, observer-blind, placebo-controlled, dose-finding trial of mRNA-1647 was conducted in CMV-seronegative and -seropositive healthy adults aged 18-40 years (NCT04232280). In Part 1, males and females were randomized 3:1 to receive mRNA-1647 (50, 100, or 150 µg) or placebo at Months 0, 2, and 6. In Part 2, females were randomized 3:1 to receive mRNA-1647 100 µg or placebo at Months 0, 2, and 6. Primary endpoints were safety throughout the study, including solicited adverse reactions (ARs) up to 7 days after each dose, and neutralizing antibody (nAb) titers against epithelial cell infection and against fibroblast infection up to 12 months after last dose. Parts 1 and 2 results are combined.

**Results:**

Of 315 adults randomized, solicited ARs following dose 1 were reported in 54.7% (placebo), 84.4% (50 µg), 87.5% (100 µg), and 91.1% (150 µg) of seronegative adults and 59.3%, 94.4%, 94.6%, and 94.4%, respectively, of seropositive adults. The most common local and systemic solicited ARs across serostatus groups after dose 1 were pain (mRNA-1647: 73.3%-89.2%; placebo: 18.5%-18.9%) and fatigue (mRNA-1647: 28.9%-72.2%; placebo: 25.9%-30.2%), respectively. Similar trends in ARs were observed after doses 2 and 3. nAb titers against epithelial cell infection and against fibroblast infection were observed in all mRNA-1647 groups (**Figure**). Robust nAb responses against epithelial cell infection were observed after each mRNA-1647 dose and sustained through the end of the study (12 months after last dose); nAb titers against epithelial cell infection in seronegative mRNA-1647 100-µg recipients exceeded the geometric mean titer of all seropositive recipients at baseline.

Antibody-Mediated Immunogenicity of mRNA-1647 by CMV Serostatus

**Figure:**
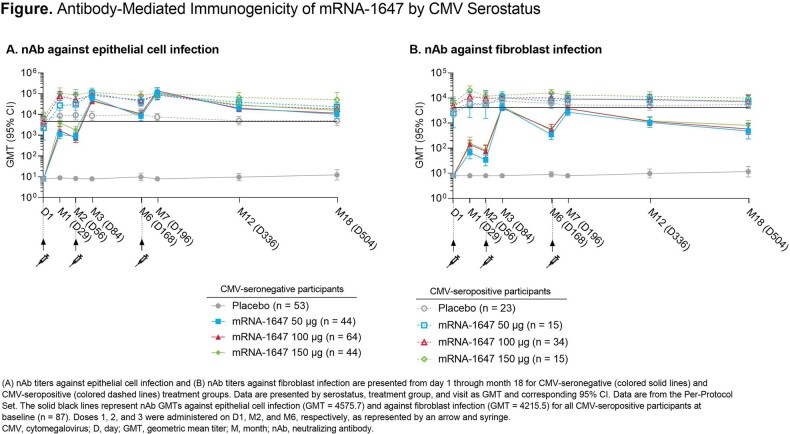

(A) nAb titers against epithelial cell infection and (B) nAb titers against fibroblast infection are presented from day 1 through month 18 for CMV-seronegative (colored solid lines) and CMV-seropositive (colored dashed lines) treatment groups. Data are presented by serostatus, treatment group, and visit as GMT and corresponding 95% CI. Data are from the Per-Protocol Set. The solid black lines represent nAb GMTs against epithelial cell infection (GMT = 4575.7) and against fibroblast infection (GMT = 4215.5) for all CMV-seropositive participants at baseline (n = 87). Doses 1, 2, and 3 were administered on D1, M2, and M6, respectively, as represented by an arrow and syringe.

CMV, cytomegalovirus; D, day; GMT, geometric mean titer; M, month; nAb, neutralizing antibody.

**Conclusion:**

mRNA-1647 was generally safe and well-tolerated and induced antigen-specific immune responses at all dose levels in both CMV-seronegative and -seropositive participants. These results guided selection of the 100-µg dose for the mRNA-1647 phase 3 trial.

**Disclosures:**

**Lori Panther, MD, MPH**, Moderna, Inc.: Employee|Moderna, Inc.: Stocks/Bonds **Sandeep Basnet, MD**, Moderna, Inc.: Employee|Moderna, Inc.: Stocks/Bonds **Richard Leggett, DO**, Crossroads Clinical Research: Contract employee **James Peterson, MD**, Moderna, Inc.: Received payment as a study investigator **Jiang Lin, PhD**, Moderna, Inc.: Employee|Moderna, Inc.: Stocks/Bonds **Kai Wu, PhD**, Moderna, Inc.: Employee|Moderna, Inc.: Stocks/Bonds **Heather Lee, BS**, Moderna, Inc.: Employee|Moderna, Inc.: Stocks/Bonds **Roxane Hasselbeck, BA**, Moderna, Inc.: Employee|Moderna, Inc.: Stocks/Bonds **Andrew Natenshon, MA**, Moderna, Inc.: Employee|Moderna, Inc.: Stocks/Bonds **Jacqueline Miller, MD**, Moderna, Inc.: Employee|Moderna, Inc.: Stocks/Bonds

